# Limitations of Jaw Movement in Fibrodysplasia Ossificans Progressiva: A Review

**DOI:** 10.3389/fmed.2022.852678

**Published:** 2022-03-22

**Authors:** Ton Schoenmaker, Amine Dahou Bouchankouk, Semih Özkan, Marjolijn Gilijamse, Elinor Bouvy-Berends, Coen Netelenbos, Frank Lobbezoo, Elisabeth Marelise W. Eekhoff, Teun J. de Vries

**Affiliations:** ^1^Department of Periodontology, Academic Centre for Dentistry Amsterdam (ACTA), University of Amsterdam, Vrije Universiteit, Amsterdam, Netherlands; ^2^Department of Oral and Maxillofacial Surgery and Oral Pathology, Amsterdam University Medical Centre (UMC), Location Free University Medical Centre (VUmc), Vrije Universiteit, Academic Centre for Dentistry Amsterdam (ACTA), University of Amsterdam, Vrije Universiteit, Amsterdam, Netherlands; ^3^Dutch Fibrodysplasia Ossificans Progressiva (FOP) Foundation and Former Centre Special Care Dentistry Rijnmond, Rotterdm, Netherlands; ^4^Department of Internal Medicine Section Endocrinology, Amsterdam University Medical Centre (UMC), Location Free University Medical Centre (VUmc), Amsterdam Movement Sciences, Amsterdam Bone Centre, Amsterdam, Netherlands; ^5^Department of Orofacial Pain and Dysfunction, Academic Centre for Dentistry Amsterdam (ACTA), University of Amsterdam, Vrije Universiteit, Amsterdam, Netherlands

**Keywords:** fibrodysplasia ossificans progressiva, maxillofacial region, jaw, maximum mouth opening, dental, review

## Abstract

Fibrodysplasia ossificans progressiva (FOP) is a rare genetic disorder characterized by heterotopic ossification (HO) of the skeletal muscles, fascia, tendons and ligaments. Patients often experience limitations in jaw function due to HO formation in the maxillofacial region. However, no studies have yet analyzed the age of onset and location of HO and the type of restrictions it may yield in the maxillofacial region. The aim of this study was to evaluate all existing literature on the site of onset of HO and associated functional restrictions of the jaw. To this end, a scoping review was performed focusing on limitations of jaw movement in FOP patients. The literature search resulted in 725 articles, of which 30 articles were included for full study after applying the exclusion criteria. From these articles 94 FOP patients were evaluated for gender, age, presence and age at which HO started in the maxillofacial region, location of HO, whether HO was caused spontaneous or traumatic and maximum mouth opening. Formation of HO is slightly more common in female patients compared to male patients, but the age of HO onset or the maximum mouth opening does not differ between genders. Trauma-induced HO occurred at a significantly younger age than spontaneous HO. Interestingly, a difference in maximum mouth opening was observed between the different ossified locations in the maxillofacial region, with ossification of the masseter muscle resulting in the smallest and ossification of the zygomatic arch resulting in the largest maximum mouth opening. This review revealed that the location of the maxillofacial region affected by HO determines the degree of limitations of the maximum mouth opening. This finding may be important for establishing clinical guidelines for the dental management of FOP patients.

## Introduction

Fibrodysplasia ossificans progressiva (FOP) is a rare autosomal dominant disorder with a prevalence of 1 in 2 million people (1, 2). It is characterized by congenital malformation of the great toes (bilateral hallux valgus) and by progressive heterotopic ossification (HO) of the skeletal muscles, fascia, tendons and ligaments (3, 4). The disease is solely caused by a single nucleotide mutation in the bone morphogenetic protein (BMP) type I receptor ACVR1 gene, located on chromosome 2 q 23-24, most frequently at nucleotide position 617, causing an arginine to histidine substitution at amino acid position 206 (R206H) (5). This mutation makes the receptor more sensitive to BMP signaling and simultaneously causes a decreased binding of the inhibitor FKBP12 to the receptor. This altered sensitivity of the receptor ultimately results in heterotopic bone formation that causes severe and irreversible movement impairments throughout the body. Recently, it was shown in two independent studies that Activin-A is the ligand that in particular causes heterotopic bone formation. It was shown in a study using induced pluripotent stem (IPS) cells carrying the mutation (6) that Activin-A induced more osteogenic signaling in FOP derived IPS cells. Simultaneously, it was shown in a mouse model that Activin-A neutralizing antibodies prevented HO (7). In the context of the maxillofacial area, we recently showed using primary periodontal ligament fibroblasts from controls and from FOP patients, that Activin A only caused differential gene expression in fibroblasts carrying the R206H mutation (8). The formation of heterotopic bone is often preceded by flare-ups characterized by local and warm swellings accompanied by pain (9). Progressive episodes of flare-ups, causing new HO formation, can lead to ankylosis of all major joints of the axial and appendicular skeleton, including the temporomandibular joints (TMJs) (10). While the age at which the first signs of FOP are noticed varies greatly, the mean age at which the first flare-ups are noticed is believed to be around 6 years old (10, 11). HO usually starts in the neck, thoracic region and back, but later in life other joints like shoulders, hips, elbows and knees are also affected, including the TMJ that allows jaw movement (3). Heterotopic bone formation can occur spontaneously or can be induced by trauma, for example after intramuscular injections, or bigger trauma such as after (heterotopic) bone excision or after dental treatment. Although the TMJ is often not the first visible joint to be affected by FOP, ~70% of the FOP patients have developed limited jaw movements by an average age of 19 years (12, 13).

The most common cause of HO in the masticatory muscles ultimately leading to TMJ deformities is trauma (14). As mentioned earlier, different kinds of trauma can induce HO in FOP patients. When looking at the temporomandibular region, overstretching the jaw during dental treatment, mandibular anesthetic blocks, and surgical trauma associated with resection of heterotopic bone can all lead to severe episodes of new bone formation (4, 12, 14, 15). In FOP the masticatory muscles, such as the masseter muscle and the pterygoid muscle, can be converted into heterotopic bone by a process called ossification. Also ankylosis can occur at bony locations in the TMJ. Both of these events can result in limitations in jaw movement and a decreased maximum mouth opening. The maximum (voluntary) mouth opening is normally measured as the distance between the mesioincisal edge of the right upper and lower central incisor tooth.

The jaw movement and maximum mouth opening can gradually decrease to zero millimeter. This has serious consequences for eating, oral hygiene, dental treatment and may even cause emetophobia, or fear of vomiting, which has been reported to considerably reduce quality of life (12, 16, 17). Possibly due to the low prevalence of the disease, comprehensive clinical dental guidelines are lacking.

Although several case report studies and some reviews describe the cause, location and impact limited jaw movement can have on individual FOP patients, there is no general overview on the etiology of limited jaw movement in FOP.

Therefore, we performed a scoping review to identify factors that are involved in limited jaw movement in FOP (18, 19). The data obtained here were used to investigate the relation between age and gender on the existence of HO in the maxillofacial region, the relation between the cause and specific location of HO and the effect on jaw movement and finally, since decrease in maximum mouth opening has a considerable effect on the QOL of FOP patients, whether the decrease in mouth opening can be attributed to a specific location of HO in the maxillofacial area.

## Materials and Methods

### Literature Search

This study has been approved by the Ethics committee of ACTA (nr. 2021-Bouchankouk). A structured methodological approach was followed by applying the PRISMA (Preferred Reporting Items for Systematic Reviews and Meta-Analyses) principles, with the aim to reduce bias in the selection of the publications. Subsequently a scoping review as described by Peters et al. (19) was performed.

Embase, PubMed and Web of Science databases were used. All publications in English up to January 2021 were included. For the search strategy the following keywords were used:

#1 fibrodysplasia ossificans progressiva OR myositis ossificans

#2 jaw OR Jawbone OR mandibular OR maxillary OR masticatory OR temporomandibular joint

#3 dentistry OR dental

#4 (#1 AND #2)

#5 (#1 AND #3)

#6 (#1 AND #2 AND #3)

The initial selection of articles was taken from the sum of #4, #5, and #6.

### Screening and Selection

Inclusion criteria were case studies clinical studies that included more than one patient demonstrating limited jaw movement in patients with fibrodysplasia ossificans progressive (FOP), and publications on dental treatment in FOP patients. Exclusion criteria were *in vitro* and animal studies and articles discussing other forms of HO, such as MOT (myositis ossificans traumatica) due to their different etiology.

Two independent reviewers screened all titles and abstracts of the publications found by the electronic search. The full text of the publication was read by both the reviewers when no similar decision by the two reviewers could be made on inclusion or exclusion or when the adequacy of the publication was questionable.

### Data Extraction and Analysis

The most common patient data such as gender, age, ankylosis/heterotopic ossification, onset of HO, location of HO in the maxillofacial region, dental treatment, maximum mouth opening, surgery and FOP flare-up were extracted from the selected studies and incorporated in Supplementary Table 1.

Independent samples *t*-tests were performed to compare the means of two variables. Chi-square tests were performed to discover coherence between two nominal variables. To determine the presence and the degree of correlation, a Pearson correlation test was performed. One-way ANOVA tests were carried out to compare distributions of multiple variables. Statistical analysis was performed with IBM SPSS statistics (version 27.0.1.0). *P*-values below 0.05 were considered statistically significant.

## Results

### Literature Search

**Figure 1:**
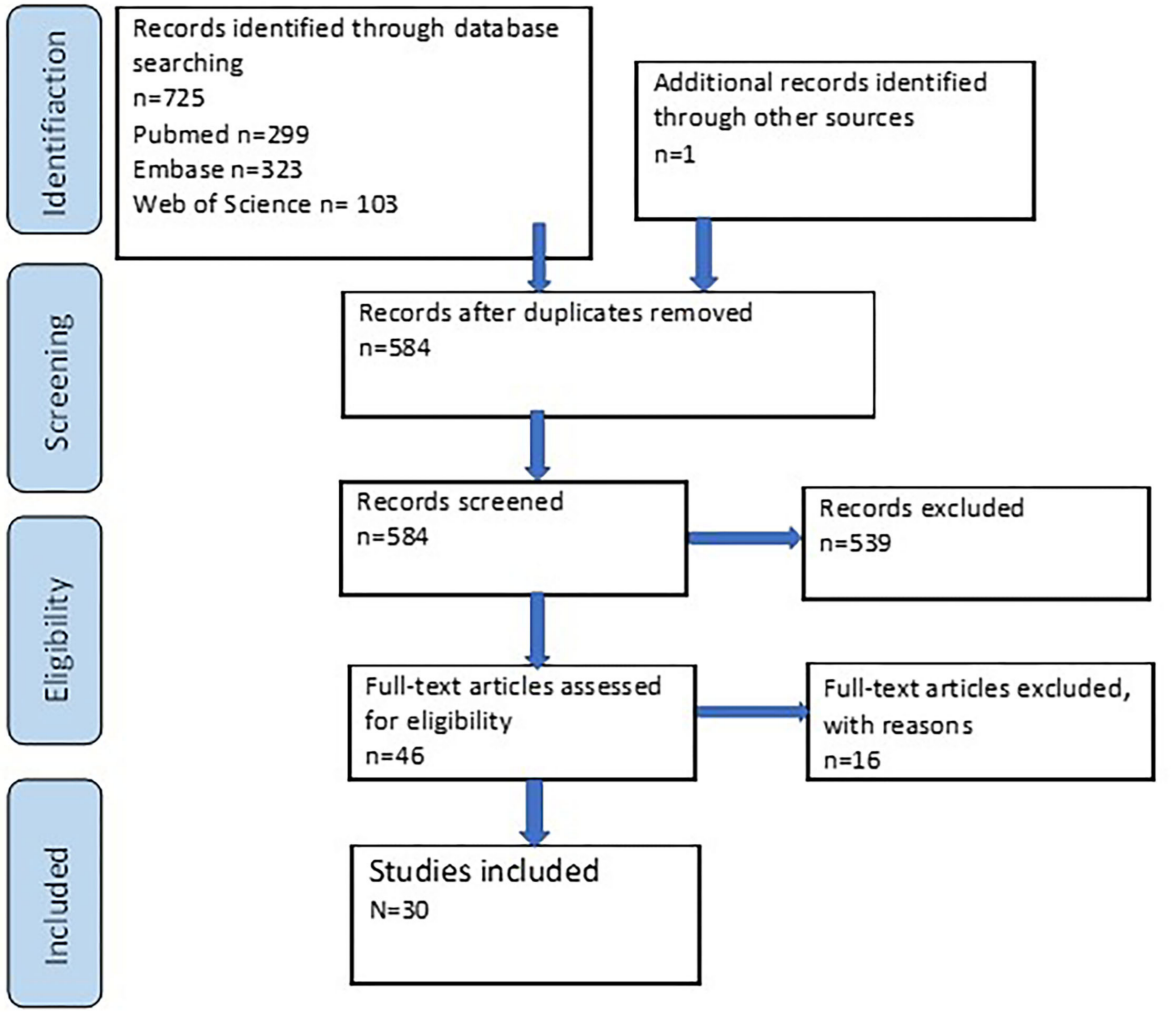
Flowchart of the literature search strategy. Records were included or excluded based on the beforehand defined criteria.

**Figure 2:**
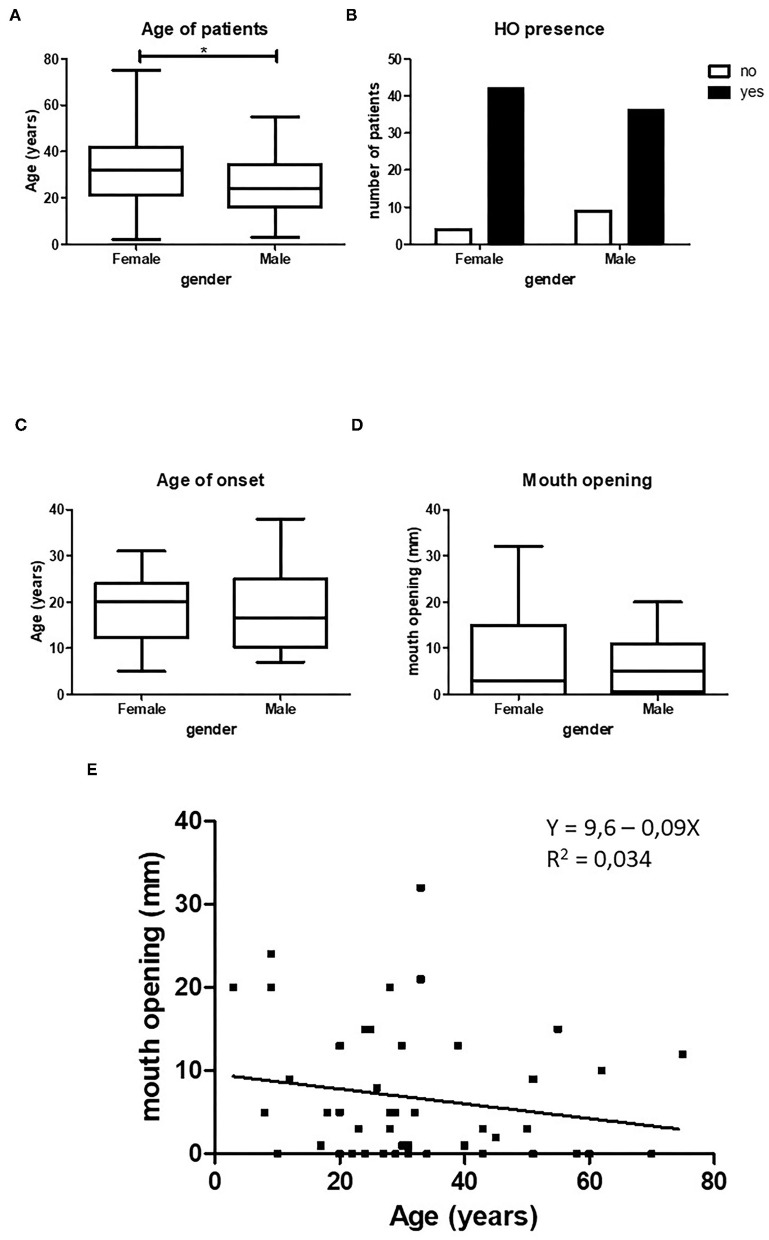
Relation between HO and gender. Female patients in our cohort were significantly older than male patients, **(A)** (*p* < 0.5, female *n* = 47, male *n* = 45). Female patients show statistically more HO in the maxillofacial area compared to male patients, **(B)** (*p* < 0.05, female *n* = 47, male *n* = 45). There is no difference between age of onset of HO (**C**, female *n* = 34, male *n* = 24) or between mouth opening (**D**, female *n* = 23, male *n* = 25) and gender. When both female and male patients are taken together there is a non-significant decrease in maximum mouth opening with increasing age (**E**, *n* = 48). *p < 0.05.

**Figure 3:**
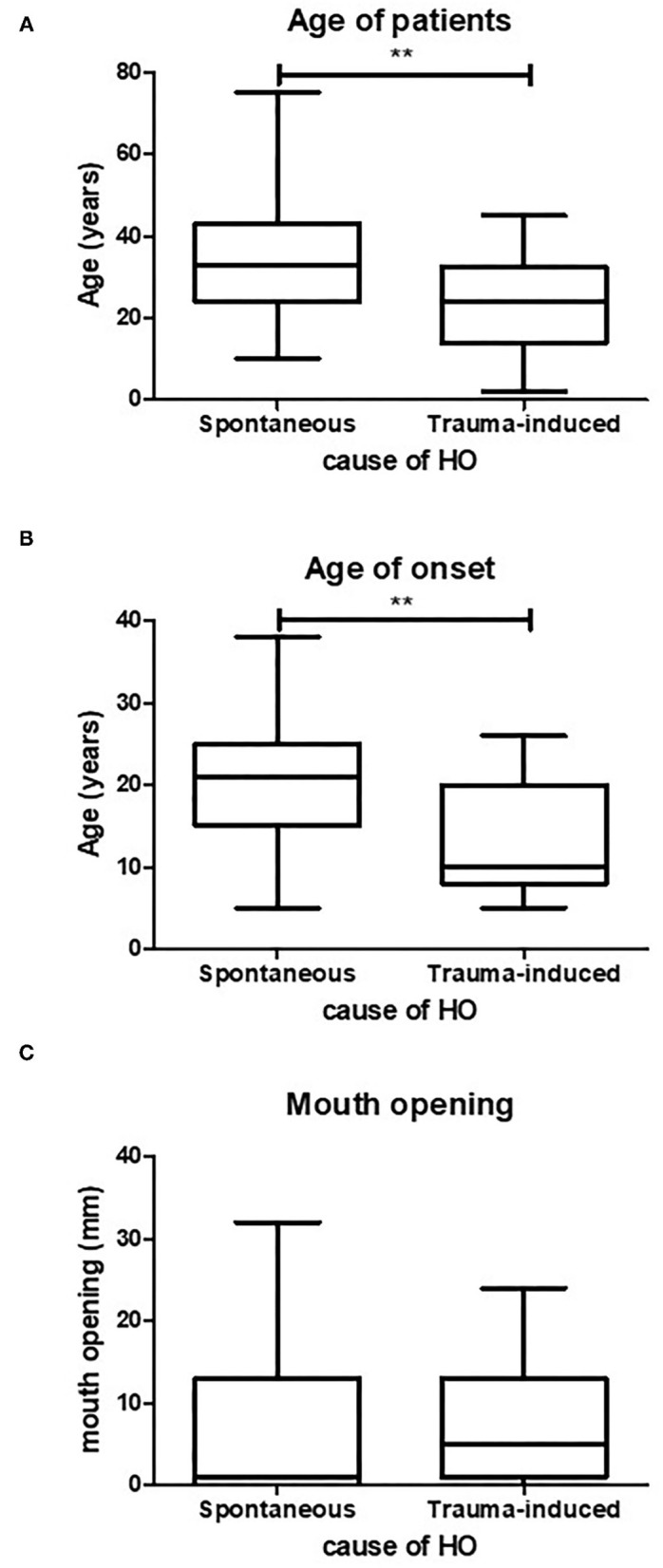
Age of spontaneous or trauma-induced HO. Trauma-induced HO occurs at a significantly lower age compared to spontaneously occurring HO, **(A)** (*p* < 0.05, spontaneous *n* = 47, trauma induced *n* = 22). Also the mean age of onset is significantly lower in trauma-induced HO, **(B)** (*p* < 0.05, spontaneous *n* = 41, trauma induced *n* = 17). There is no difference in maximum mouth opening between the two causes of HO **(C)** (spontaneous *n* = 27, trauma induced *n* = 11). **p < 0.01.

The search of all databases together resulted in 725 papers, of which 142 papers were duplicates. One additional paper was added after a manual search in the reference list of an unpublished guideline on medical management of FOP patients. Screening of titles and abstracts of the 584 identified papers were performed individually by the two reviewers (AD, SO), resulting in 539 publications that were excluded because they did not meet the inclusion criteria. Of the remaining 46 papers, an additional 16 were excluded either because there was no full text available or because, after reading the full texts, no relevant data could be extracted from the article. Ultimately, the search and selection resulted in the inclusion of 30 articles (12, 15–17, 20–45) (Figure 1). These articles were published between 1982 and 2020. The included studies and associated data can be found in Supplementary Table 1. The data shown in Supplementary Table 1 formed the basis for the Figures 2, 3, **5** presented in the manuscript.

### Influence of Gender on HO

The total number of patients with HO in the maxillofacial region retrieved from the 30 case studies was 94. Exactly half of the patients were female. The average age was 29.5 years (ranging from 2 to 75 years). The female patients were significantly older than male patients (Figure 2A). HO in the maxillofacial region was more frequent in female patients (Figure 2B).

The mean age at onset of HO in the maxillofacial region of the entire patient population was 18.1 years (±7.9 years). Although the female patients were older than the male patients, no significant difference was found between the mean age of onset between the two groups (Figure 2C). The mean maximum mouth opening was not found to be different between female and male patients (Figure 2D).

Since we found no significant differences in maximum mouth opening between the sexes, we pooled the two groups and assessed whether there was a correlation between age and maximum mouth opening. Figure 2E shows an inverse relation between mouth opening and age, but this correlation was not significant.

### Trauma-Induced HO in the Maxillofacial Region Occurs at an Earlier Age Than Spontaneous HO

As discussed earlier, HO formation in FOP patients can be triggered by several causes. Clinically, the causes of HO are divided into spontaneous or trauma-induced. Spontaneously induced HO arises with no known recollection of trauma to the jaw. The trauma-induced group includes all kinds of traumas to the jaw, such as fall trauma, bone excision, intramuscular injections, dental treatment and surgeries that likely resulted in HO in the maxillofacial region. To investigate whether there is a relation between age of onset and the causes of HO, and whether the cause of HO predicts the maximum mouth opening, these parameters were tested on the pooled data. Patients with trauma-induced HO were more than 10 years younger with a mean age of 24.8 years than patients with spontaneous HO who had a mean age of 35.6 years (Figure 3A). Also, as shown in Figure 3B, the mean age at which trauma induced HO occurred was significantly lower (13 years) than that of spontaneously occurring HO (20 years).

Despite the fact that trauma-induced HO occurs at a younger age compared to spontaneously occurring HO, the mean maximum mouth opening did not differ between the two groups (Figure 3C).

### Sites of HO in the Maxillofacial Region

**Figure 4:**
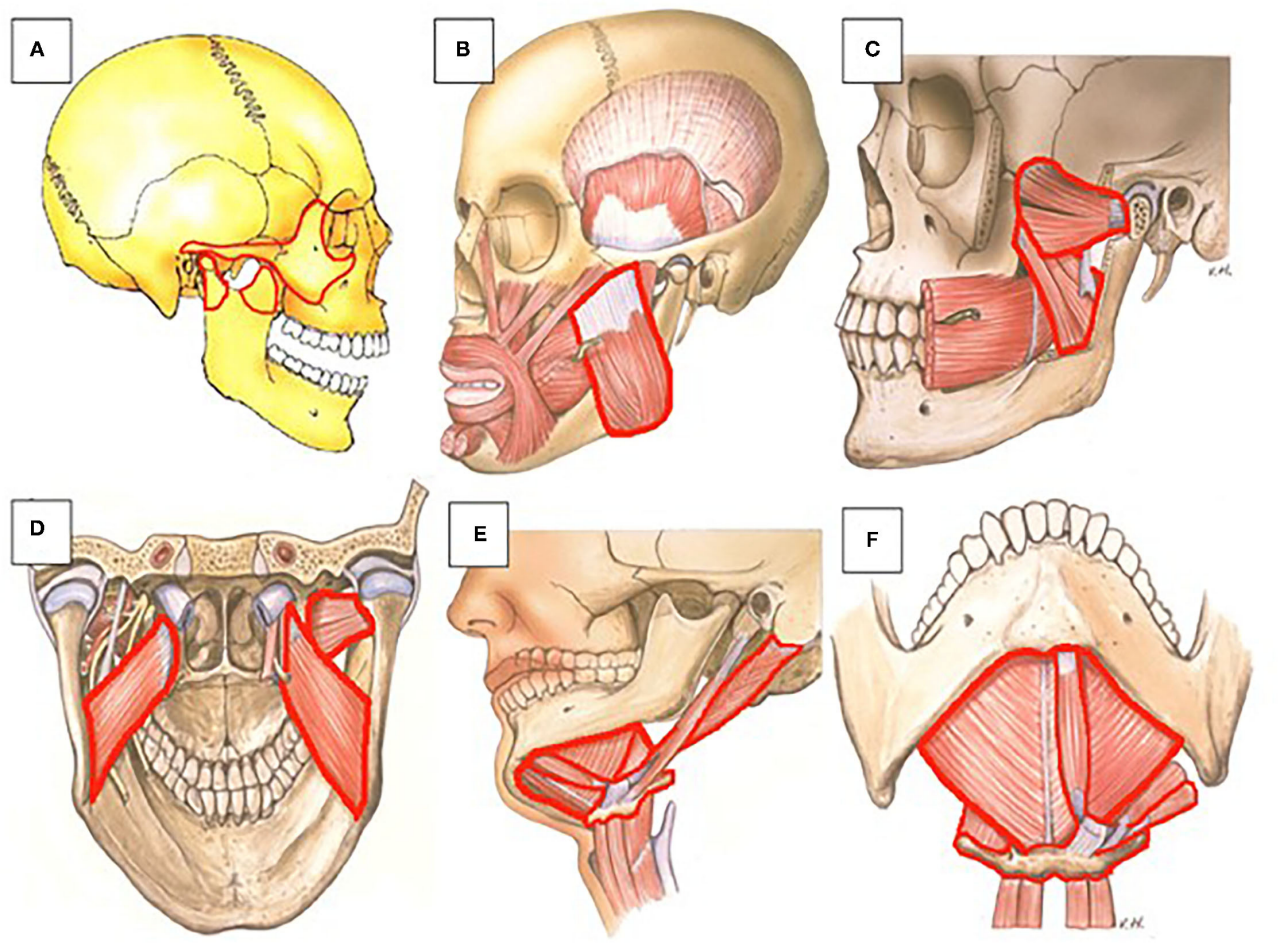
Anatomic representation of maxillofacial structures and muscles that can be affected by HO in FOP patients. **(A)** (1) The coronoid process (front side of the rami) can ossify with the zygomatic arch. (2) The coronoid process and zygomatic arch can ossify individually. (3) The condyle process (back side of the rami) can ossify individually (lateral view). **(B)** The masseter (lateral view). **(C)** The lateral and medial pterygoid (lateral view). **(D)** The lateral and medial pterygoid (posterior view). **(E)** The suprahyoid muscles (digastric, geniohyoid, and mylohyoid) can ossify with the hyoid bone (lateral view). **(F)** The suprahyoid muscles (digastric, geniohyoid, and mylohyoid) can ossify with the hyoid bone (inferior view). Frequencies of HO of the various muscles and bony regions are shown in Figure 5. Picture: Courtesy of Jan Harm Koolstra, ACTA, The Netherlands.

HO in the maxillofacial region may occur in some limited locations. Sites of ankylosis and/or HO described in the 30 studies were the zygomatic arch (8.3%, *n* = 2), the coronoid process (12.5%, *n* = 3), from the zygomatic arch to the coronoid process (16.7%, *n* = 4), the pterygoid muscle (33.3%, *n* = 8, 7 of which describe ossification of the lateral pterygoid muscle), the masseter muscle (16.7%, *n* = 4), the condyle head (4.2%, *n* = 1) and from the mentum to the hyoid bone (8.3%, *n* = 2) (Supplementary Table 1; Figure 4). Due to the low number of cases per location no statistics could be performed on these data. They will be discussed as tendencies below.

**Figure 5:**
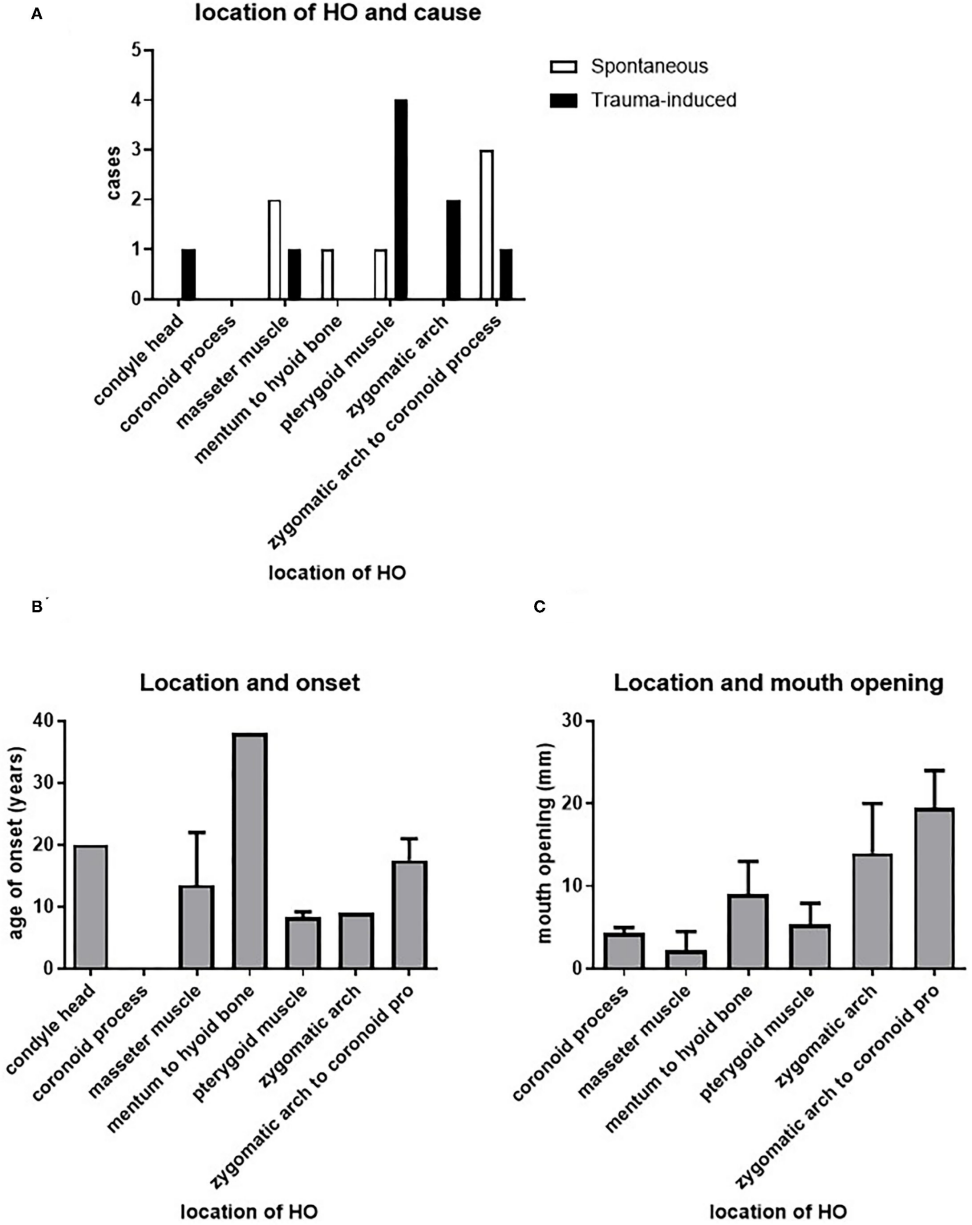
Relation between HO and location. There does not seem to be a correlation between the cause of HO or the age of onset of HO and its location **(A,B)**. The different possible locations of HO seem to differ in their effect on maximal mouth opening, with a significant difference between HO in the masseter muscle and HO between the zygomatic arch and the coronoid process **(C)**.

We assessed whether there is a relation between the spontaneously or trauma-induced onset HO and the different maxillofacial sites where HO has been described. Figure 5A shows that such a relationship does not exist, although this may be due to the limited information we have on this particular topic.

We next assessed the possible relation between the age and onset of HO and the different sites affected by HO. Figure 5B shows that the pterygoid muscles and the zygomatic arch are affected at a relatively younger age, with a mean age of 8.3 and 9 years, respectively. The masseter muscle was affected at a mean onset of 13.5 years, while HO from the zygomatic arch to the coronoid process occurred at a mean onset of 17.5 years. The latest affected areas are the condylar head and from the mentum to the hyoid bone with mean onset of, respectively, 20 and 38 years.

Investigating the relationship between the maximum mouth opening and the location of HO, it was found that HO at the masseter muscle corresponded to the smallest maximum mouth opening, with a mean opening of 2.3 mm. The second smallest maximum mouth opening was found in patients with HO at the coronoid process, with a mean opening of 4.3 mm. Third smallest maximum mouth opening could be found in patients with HO at the level of the (lateral) pterygoid muscle, with a mean 5.3 mm. Patients with HO from the mentum to the hyoid bone showed a slightly larger mean of maximum mouth opening of 9 mm. HO at the zygomatic arch and at the zygomatic arch to the coronoid process showed the largest mean of maximum mouth opening in patients with means of 14 and 19.5 mm, respectively (Figure 5C). These data show that the resulting maximum mouth opening after HO may depend on the originally affected location.

## Discussion and Conclusion

In this study we reviewed 30 articles containing data on a total of 94 patients with reported limitations in their jaw movement. We focused especially on HO formation in the maxillofacial region, as this often leads to this limited jaw movement and can result in reduced maximum mouth opening.

The etiology of limited jaw movement in FOP has not been studied extensively before. Here, we aimed to study possible relationships between mouth opening and HO in the maxillofacial region with age and gender, the cause of HO and the anatomical location where HO arose. Since articles were selected based on reported limitations of jaw movement, our study focuses only on these reports. Etiology and progression of limitations on jaw movement were drawn solely from these included articles with reported and measured jaw restrictions. Therefore, no data were retrieved on the occurrence of jaw restrictions, albeit that in daily clinical practice this is extremely common in FOP patients.

Female and male patients were equally represented in the patient population, which is in accordance with previous studies identifying FOP as an autosomal disease where both genders are affected equally (4, 10). The female patients were significantly older than the male patients in this study. They were also more frequently affected by HO in the maxillofacial region, which could be due to the relatively higher mean age of this group. There is a tendency toward an inverse correlation between age and maximum mouth opening, which is to be expected given the progressive nature of FOP (3, 10). We observed a significant difference in the age at which HO was present in the maxillofacial region between the patients with HO that occurred spontaneously and the patients with trauma-induced HO. Trauma-induced HO occurred at a significantly younger age than spontaneous HO. This could be due to more frequent dental interventions because (FOP) children tend to visit the dentist more frequent compared to older patients. In addition, dento-alveolar traumas are often caused by playing and falling. There may also be a relationship with orthodontic treatments which are occasionally performed in FOP patients and could possibly cause trauma that could lead to HO. Although, according to the current medical management of the international clinical council on FOP, orthodontic treatment can be safely performed, insight in the relation between the applied forces and tooth movement is lacking. Quite possibly such forces on muscles and ligaments finally could induce HO in that area. The reviewed articles in this study did not provide enough information on what type of trauma actually induced the HO to draw any further conclusions on this.

Interestingly, differences were found between the maximum mouth opening and the different locations affected by HO. HO located at the coronoid process (Figure 4A) and at the masseter (Figure 4B) and pterygoid muscles (Figures 4C,D) corresponded with the smallest maximum mouth opening. A possible explanation could be that the masseter muscle mainly functions as a powerful elevator of the mandible. Ossification of this muscle could lead to decreased mouth opening. There are two types of pterygoid muscles, the medial and the lateral. The medial pterygoid muscle mainly functions as an elevator of the mandible, albeit not as powerful as the masseter muscle. In all but one of the report cases used in this review, ossification of the lateral pterygoid is described. In the report were ossification of the medial pterygoid is described this ossification is compared to two other cases where a bony bridge is described between the mentum of the mandible and the hyoid bone formed by ossification of the suprahyoid muscles that act as mandibular depressors (25). This bony bridge resulted in a much smaller maximum mouth opening compared to the HO in the medial pterygoid. Therefore, Okuna et al. suggested that the effect on maximum mouth opening could be bigger when depressors of the mandible are affected compared to affected elevators. Our data, however, suggest otherwise since ossification of the masseter, a powerful elevator, seems to result in the smallest maximum mouth opening compared to other locations.

The lateral pterygoid muscle on the other hand has an opposite role and mainly functions as a depressor of the mandible. As mentioned, in all but one of the articles included in this review where the pterygoid muscles were involved, HO was located in the lateral pterygoid. In the majority of these cases ossification of the lateral pterygoid results in a bony connection between the lateral pterygoid plate and the mandibular ramus. Also, the deep part of the masseter muscle and the lateral pterygoid muscle both have fibers that connect to the articular disc of the TMJ and both muscles are responsible for protrusion of the jaw (46). Since protrusion and rotation of the condyle head of the TMJ is required for opening the jaw, HO at these sites could lead to greater limitations of jaw movement and thus has a big impact on the final maximum mouth opening. Another disease characterized by HO is Myositis Ossificans Traumatica (MOT). Also in MOT limitations of jaw movement and reduced maximum mouth opening have been reported because of HO in the maxillofacial region, again involving the masseter and/or the pterygoid muscles in most cases (47). Taken together, this implies that ossification of the muscles that elevate or repress the mandible can both result in reduced mouth opening.

A limitation of this study is the low prevalence of FOP and the resulting limited number of articles on FOP and jaw related functional restrictions. Not all parameters of the patients were given and discussed in the available studies. In addition, the way in which maximum mouth opening was measured was not discussed in all case studies, possibly resulting in clinical heterogeneity on these data.

Malnutrition and starvation without external supplements can occur with a reduced maximum mouth opening. The average maximum mouth opening in the normal population is 45 mm (female) and 50 mm (male). When the maximum mouth opening drops below 20 mm, as is the case in many FOP patients, it poses problems with oral hygiene, leaving FOP patients prone to dental caries and other dental problems while dental treatment is difficult due to limited access to the mouth (4, 25). In addition, dental treatments which require a mandibular block anesthesia and stretching of the jaw can cause or exacerbate heterotopic ossification (12, 14, 15). This review suggests that trauma, especially in the masseter muscle and the pterygoid muscles, should be avoided since HO at these sites results in the smallest maximum mouth opening and thus has the biggest impact on FOP patients. A mandibular block anesthesia in FOP patients is contraindicated, as it can lead to ossification of the pterygoid muscles which can cause ankylosis of the TMJ (14, 15). Furthermore, it is advised to avoid forced pressure on the jaw during medical or dental treatment as this can lead to HO in the maxillofacial region and to severely reduced maximum mouth opening.

## Author Contributions

TS designed the study and wrote the manuscript. AD and SÖ conducted the review. MG, EB-B, CN, and FL contributed to the design of the study and writing the manuscript. EE and TV supervised the study, contributed to the study design and writing the manuscript. All authors contributed to the article and approved the submitted version.

## Conflict of Interest

The authors declare that the research was conducted in the absence of any commercial or financial relationships that could be construed as a potential conflict of interest.

## Publisher's Note

All claims expressed in this article are solely those of the authors and do not necessarily represent those of their affiliated organizations, or those of the publisher, the editors and the reviewers. Any product that may be evaluated in this article, or claim that may be made by its manufacturer, is not guaranteed or endorsed by the publisher.
