# Supplementary material for: Limitations of Jaw Movement in Fibrodysplasia Ossificans Progressiva: A Review
Source: Front Med (Lausanne). 2022 Mar 22;9:852678. doi: 10.3389/fmed.2022.852678 (PMC8980542; doi:10.3389/fmed.2022.852678)
Supplement: Supplementary file 1 [file Table_1.pdf]

Supplementary Table 1

Data obtained from the 94 described patients.

Author	Sex	Age (years)	HO maxillofacial	Onset HO maxillofacial (years)	Location HO	Spontaneous (S) or trauma (T)	Abnormalities maxillofacial
Renton et al. 1982	M	7	N	-	-	-	The condyles were broad and the articular surfaces flattened.
	M	4	N	-	-	-	The condyles were broad and the articular surfaces flattened.
Connor et al. 1982	F	25	Y	21	-	S	-
	F	28	Y	25	-	S	-
	F	60	Y	15	-	S	-
	F	58	Y	20	-	S	-
	M	31	Y	12	-	T	-
	M	10	Y	10	-	S	-
	M	20	Y	12	-	S	-
	M	27	Y	24	-	S	-
	F	29	Y	18	-	S	-
	F	33	Y	24	-	S	-
	F	30	Y	15	-	S	-
	M	30	Y	26	-	T	-
	M	28	Y	11	-	T	-
	F	34	Y	14	-	T	-
	F	33	Y	25	-	S	-
	M	20	Y	8	-	T	-
	M	31	Y	7	-	S	-

	F	70	Y	21	-	S	-
	M	51	Y	25	-	S	-
	F	24	Y	5	-	S	-
	F	26	Y	20	-	T	-
	F	34	Y	26	-	S	-
	F	40	Y	23	-	S	-
Crofford et al. 1990	M	-	Y	21	Coronoid process to zygomatic arch	S	-
	M	-	Y	15.5	M.pterygoid	S	-
Luchetti et al. 1996	F	22	Y	9	-	T	-
	M	35	Y	18	-	S	-
	F	36	Y	24	-	S	-
	F	33	Y	13	-	S	-
	F	44	Y	10	-	T	-
	F	56	Y	24	-	S	-
	F	46	Y	23	-	S	-
	F	35	Y	24	-	S	-
	M	15	N	-	-	-	-
	M	41	Y	12	-	S	-
	M	45	Y	32	-	S	-
	F	54	Y	31	-	S	-
	M	41	Y	15	-	S	-
	M	33	Y	33	-	S	-
	F	32	Y	26	-	S	-
	F	22	Y	19	-	S	-
	M	25	Y	25	-	S	-
	F	36	Y	20	-	T	-
	F	14	Y	7	-	T	-

	F	42	Y	26	-	T	-
	F	40	Y	15	-	S	-
	M	9	N	-	-	-	-
	M	34	Y	25	-	S	-
	M	7	N	-	-	-	-
	M	22	N	-	-	-	-
	M	20	N	-	-	-	-
	M	20	N	-	-	-	-
	M	12	Y	9	-	S	-
	M	24	Y	18	-	S	-
	F	11	Y	7	-	S	-
	F	20	N	-	-	-	-
	F	21	N	-	-	-	-
	F	69	Y	26	-	S	-
Chichareon et al. 1999	M	3	-	-	-	-	-
Herford et al. 2003	M	24	Y	14	Coronoid process to zygomatic arch	S	-
Sendur et al. 2006	F	20	Y	-	-	S	-
Vashisht et al. 2006	F	12	Y	5	M.masseter	T	-
Van der Meij et al. 2006	F	9	Y	9	Zygomatic arch	T	-
Young et al. 2007	F	24	Y	22	M.masseter	S	-
Wadenya et al. 2010	M	20	Y	20	Condyle process	T	-
Duan et al. 2010	M	17	Y	-	M.pterygoid	-	-
Carvalho et al. 2010	M	13	Y	-	M.pterygoid	-	The condyles were broad and the articular surfaces flattened. Retrognathia.
Carvalho et al. 2011	M	13	Y	7	M.pterygoid	T	The condyles were broad and the articular surfaces flattened. Retrognathia.
	F	21	Y	10	M.pterygoid	T	The condyles were broad and the articular surfaces flattened. Retrognathia.

	M	22	Y	8	M.pterygoid	T	The condyles were broad and the articular surfaces flattened. Retrognathia.
Mori et al. 2011	M	18	Y	-	Coronoid process	-	The condyles were broad and the articular surfaces flattened. Retrognathia.
Roberts et al. 2011	F	43	Y	-	M.masseter	S	-
	M	43	Y	-	-	S	-
	F	6	N	-	-	-	Hypoplastic mandible.
	F	75	Y	-	-	S	-
	F	2	-	-	-	T	Hypoplastic mandible.
Braga et al. 2011	F	23	Y	-	-	S	-
Susami et al. 2012	M	8	Y	-	Coronoid process	-	The condyles were broad and the articular surfaces flattened.
Orhan et al. 2012	F	20	Y	-	Coronoid process to zygomatic arch	S	The condyles were broad and the articular surfaces flattened.
Mortazavi et al. 2012	M	28	Y	-	-	-	-
Kriegbaum et al. 2013	M	26	Y	-	Zygomatic arch	-	-
Haupt et al. 2016	M	22	Y	-	M.masseter	-	-
Okuno et al. 2017	F	29	Y	-	Mentum to hyoid bone	-	-
	M	39	Y	38	Mentum to hyoid bone	S	-
	F	62	N	-	M.pterygoid	-	-
Eekhoff et al. 2018	F	9	Y	-	Coronoid process to zygomatic arch	T	-
El Azem et al. 2018	M	55	N	-	-	-	-
Geddis Regan et al. 2018	M	45	-	-	-	T	The condyles were broad and the articular surfaces flattened.
Rajanikanth et al. 2018	M	32	Y	-	M.pterygoid	T	-
Dutra et al. 2019	F	32	N	-	-	T	-
Deguchi et al. 2020	M	51	Y	-	-	-	-

Leavitt et al. 2009	M	50	Y	-	Coronoid process	-	The right condylar head had irregular borders.
